# Computation of soliton structure and analysis of chaotic behaviour in quantum deformed Sinh-Gordon model

**DOI:** 10.1371/journal.pone.0304424

**Published:** 2024-06-21

**Authors:** Adil Jhangeer, Farheen Ibraheem, Tahira Jamal, Muhammad Bilal Riaz, Atef Abdel Kader

**Affiliations:** 1 IT4Innovations, VSB - Technical University of Ostrava, Poruba, Ostrava, Czech Republic; 2 Department of Mathematics, Namal University, Mianwali, Pakistan; 3 Department of Mathematics, Forman Christian College-A Chartered University-FCCU, Lahore, Pakistan; 4 Department of Mathematics, University of the Punjab, Lahore, Pakistan; 5 Department of Computer Science and Mathematics, Lebanese American University, Byblos, Lebanon; 6 College of Humanities and Sciences, Ajman University, Ajman, UAE; Tel Aviv University, ISRAEL

## Abstract

Soliton dynamics and nonlinear phenomena in quantum deformation has been investigated through conformal time differential generalized form of *q* deformed Sinh-Gordon equation. The underlying equation has recently undergone substantial amount of research. In Phase 1, we employed modified auxiliary and new direct extended algebraic methods. Trigonometric, hyperbolic, exponential and rational solutions are successfully extracted using these techniques, coupled with the best possible constraint requirements implemented on parameters to ensure the existence of solutions. The findings, then, are represented by 2D, 3D and contour plots to highlight the various solitons’ propagation patterns such as kink-bright, bright, dark, bright-dark, kink, and kink-peakon solitons and solitary wave solutions. It is worth emphasizing that kink dark, dark peakon, dark and dark bright solitons have not been found earlier in literature. In phase 2, the underlying model is examined under various chaos detecting tools for example lyapunov exponents, multistability and time series analysis and bifurcation diagram. Chaotic behavior is investigated using various initial condition and novel results are obtained.

## Introduction

Nonlinear models have earned considerable amount of attention due to their potential of demonstrating the dynamics of numerous natural and scientific phenomena, such as plasma waves (acoustic), gravitational waves, shallow water dynamics, fluid dynamics, nonlinear optics, and surface ocean waves. [1–6]. Nonlinear partial differential equations (NLPDE) are essential for simulating exciting phenomenon in these areas and numerous real-world problems. Analytic solution of NLPDE, therefore, have been well researched, ever evolving and challenging field of exploration. Some eminent contributions are [7–12].

Quantum physics that regulates microscopic systems is a best example of the *q* deformed Sinh-Gordon equation [13]. Numerous partial differential equations in classical calculus that have important applications in numerous fields have been studied by a number of scholars [14–16]. These models play an instrumental role both in applied sciences and Mathematics.

With the advent of fractional calculus, it is now possible to explore mathematical models in a novel manner that incorporates fractional derivatives [17–20]. Many scientists and researchers began integrating all the principles and relationships that are seen in fractional and traditional calculus into *q* calculus [21, 22]. Quantum calculus also referred as calculus without limits, is identical to conventional infinitesimal calculus but does not include the concept of limits. It provides ideas of *h*-calculus and *q* calculus where h refers Plank’s constant and *q* representing quantum [23, 24]. Relativity theory, special functions, mathematical physics along with other areas have witnessed instrumental role of *q* calculus [25–27] and partial differential equations generated in *q* calculus are termed a *q*-deformed equations.

In this research, the following generalized *q* deformed Sinh-Gordon equation (Eleuch equation) [28] is taken into investigation.

$$\begin{array}{c} \frac{\partial^{2}n}{\partial x^{2}}-\frac{\partial^{2}n}{\partial t^{2}}={\left[sinh_{q}\left(n^{\gamma }\right)\right]}^{p}-\delta , \end{array}$$ (1)

where the sinh_*q*_ function is defined by

$$\begin{array}{c} {\text{sinh}}_{q}\left(x\right)=\frac{e^{x}-qe^{-x}}{2},\;\;0<q\leq 1. \end{array}$$ (2)

For *q* = 1, Eq (2) gives typical sinh functions. The tanh_*q*_(*y*), cosh_*q*_(*y*) and their reciprocal are thoroughly explained along with their key properties in [28, 29]. Some significant contribution to the underlying problem are [30–32].

Presently, theory of chaos and bifurcation is extensively used in examining differential equations. These are typically useful tools for understanding any intricate system that differential equations may control. A bifurcation is a qualitative shift driven by parameter change in the behaviour of a dynamical system. A variety of bifurcation schemes exist, including the saddle node bifurcation, Hopf bifurcations, period doubling bifurcation and pitchfork bifurcation. Examining the complex behaviour of nonlinear waves and investigating chaos theory are integral components of studying differential equation. The investigation of Chaos, a measure of stability when an external force is introduced to a nonlinear system, is essential in this contemporary era. Autonomous dynamic system’s asymptotic behaviour is exclusively dictated by their introductory conditions. There are the four types of equilibrium behaviour: an equilibrium point, a limit cycle, a torus and chaos.

Recently, Jamal et al. [33] have studied the Novikov Veselov equation through bifurcation and chaos discovery tools and they also achieved the soliton solutions. Rafiq et al. [34] have investigated the shallow water waves through bifurcation and chaos analysis and also acquired the multi solitons. The conformable Fokas Lenells model has been examined by Lie and Huang [35], employing chaos theory and bifurcation analysis. Zhang et al. [36] carried out a investigation of bifurcation on the modified FitzHugh Nagumo neuron model and find out the novel results. Jamal et al. [37] have explored the nerve impulse model by practicing the phase portraits, quasi periodic, multistability, time series and sensitivity analysis, also obtained soliton solutions.

Liu and Li [38] examined the fractional perturbed Gerdjikov–Ivanov equation. Their research delved into the chaotic behavior of the model by introducing disturbance factors into the planar dynamical system. They analyzed various aspects of the model, including two-dimensional and three-dimensional phase portraits, Poincaré sections, and sensitivity analysis. Gu et al. [39] explored the (3+1)-dimensional negative-order Korteweg-de Vries-alogeroBogoyavlenskii-Schiff (KdV-CBS) equation, which extends the classical Korteweg-de Vries (KdV) equation and broadens the scope of nonlinear partial differential equations.

The aim of this article is to employ the new extended direct algebraic approach and modified auxiliary equation method to derive analytical solutions for the given model. As far as we are aware, this methods have not been used to evaluate Eq (1) in earlier research. The proposed approaches provides findings in several general and explicit form, covering trigonometric, exponential, rational and hyperbolic functions. This gives them various advantages over previously investigated techniques. Travelling wave transformation is used to convert nonlinear partial differential equation into nonlinear ordinary differential equation. The results are represented graphically as 3D and 2D charts for particular values of pertinent parameters. There are various methods for determining chaos [40]. In this study, the most beneficial ones are emphasised. Here are Lyapunov exponents, multistability, time series and bifurcation diagram are discussed to review the chaotic behavior of the considered model. The study, in the opinion of the authors, is intriguing and has never been presented before the system in question.

The paper has been organised according to the format provided below. The mathematical framework and computation of soliton solution of the examined model have been described in Segment material and methods. Graphical illustrations and the chaotic behavior is investigated in Segment results and discussions. The research findings are highlighted in conclusion section.

## Materials and methods

### The mathematical framework of the model

The subsequent transformation is proposed for the anatomization of the traveling wave solution of Eq (1):

$$\begin{array}{c} \eta =\frac{x-\alpha t}{\sqrt{1-{\left(\alpha \right)}^{2}}}, \end{array}$$ (3)

where the symbol *α* represents the speed of the travelling wave. Consequently, plugging Eq (3) into Eq (1), below mentioned ordinary differential equation has been derived.

$$\begin{array}{c} \frac{d^{2}n\left(\eta \right)}{d\eta^{2}}={\left[{\text{sinh}}_{q}\left(n^{\gamma }\left(\eta \right)\right)\right]}^{p}-\delta . \end{array}$$ (4)

In this article, Eq (4) will be examined for *δ* = 0, *p* = 1, and *γ* = 1 and

$$\begin{array}{c} m\left(\eta \right)=e^{n}\left(\eta \right), \end{array}$$ (5)

use Eq (5) to transform Eq (4) as:

$$\begin{array}{c} -2m^{\prime 2}+2mm^{\prime \prime }-m^{3}+qm=0. \end{array}$$ (6)

## Computation of soliton solutions for Eq (6)

### Solutions by employing the extended direct algebraic method

In this segment, the generalized *q* deformed Sinh-Gordon equation is analyzed employing extended direct algebraic strategy. The method can be realized by considering the nonlinear partial differential equation stated below:

$$\begin{array}{c} P(p,p_{x},p_{t},p_{y},p_{xx},p_{yy},p_{tt},p_{xt},\ldots )=0, \end{array}$$ (7)

where *P* is a polynomial involving higher order partial derivatives and nonlinear components in *p*(*x*, *y*, *t*). The travelling wave is transformed by a process known as:

$$\begin{array}{c} p\left(x,y,t\right)=\text{ln}\left(m\left(\eta \right)\right),\;\;\eta =\frac{x+y-\alpha t}{\sqrt{1-{\left(\alpha \right)}^{2}}}, \end{array}$$ (8)

here, *α* represents a nonzero constant. Eq (7) is transformed into a nonlinear ordinary differential equation of the following form:

$$\begin{array}{c} L(m,m^{\prime },m^{\prime \prime },\ldots )=0. \end{array}$$ (9)

In Eq (9), the prime denotes the derivative with respect to *η*. Assume that Eq (9) has a solution of the following form:

$$\begin{array}{c} m\left(\eta \right)=\sum_{i=0}^{Q}(a_{i}P{\left(\eta \right)}^{i}), \end{array}$$ (10)

where

$$\begin{array}{c} P^{\prime }\left(\eta \right)=ln\left(\rho \right)(\mu +\nu P\left(\eta \right)+\zeta P^{2}\left(\eta \right)),\;\;\;\rho \neq 0,1, \end{array}$$ (11)

while *ζ*, *ν* and *μ* are real constants, the value of *Q* can be determined by balancing the nonlinear terms and the highest order derivative appearing in Eq (9).

The following are generic solutions to Eq (11) for the parameters *ζ*, *ν* and *μ*, where Φ = (*ν*^2^ − 4*μζ*). For further information, see [41, 42].

The value of *Q* is firstly obtained using homogeneous balance principle method. The value of *Q* = 2 is achieved by balancing the nonlinear terms with the highest order derivatives in Eq (6), which ultimately implies the solution in the known form:

$$\begin{array}{c} m\left(\eta \right)=a_{0}+a_{1}P\left(\eta \right)+a_{2}P{\left(\eta \right)}^{2}, \end{array}$$ (12)

where *P*(*η*) satisfies Eq (11). Comparing the different powers of coefficients *P*(*η*), after plugging Eq (12) with Eq (11) into Eq (6) and have a system of suitable algebraic equations:

$$\begin{array}{c} \begin{array}{cc} P^{0}\left(\eta \right):\; & 2\;\text{ln}\;{\left(\rho \right)}^{2}\mu^{2}(2a_{0}a_{2}-a_{1}^{2})+2a_{0}a_{1}\;\text{ln}\;{\left(\rho \right)}^{2}\mu \nu -a_{0}^{3}+qa_{0}=0,\\ P^{1}\left(\eta \right):\; & 2\;\text{ln}\;{\left(\rho \right)}^{2}(2\zeta \mu a_{0}a_{1}-2a_{2}a_{1}\mu^{2}+6a_{2}a_{0}\mu \nu -a_{1}^{2}\mu \nu +\nu^{2}a_{0}a_{1})-3a_{0}^{2}a_{1}+qa_{1}=0,\\ P^{2}\left(\eta \right):\; & 2\;\text{ln}\;{\left(\rho \right)}^{2}(8\zeta \mu a_{0}a_{2}+3\zeta \nu a_{0}a_{1}-2a_{2}^{2}\mu^{2}-a_{1}a_{2}\mu \nu +4a_{0}a_{2}\nu^{2})-3a_{2}a_{0}^{2}\\ & -3a_{0}a_{1}^{2}+a_{2}q=0,\\ P^{3}\left(\eta \right):\; & 2\;\text{ln}\;{\left(\rho \right)}^{2}(2a_{0}a_{1}\zeta^{2}+2\zeta \mu a_{2}a_{1}+10\zeta \nu a_{2}a_{0}+\zeta \nu a_{1}^{2}-2\mu \nu a_{2}^{2}+a_{2}a_{1}\nu^{2})\\ & -6a_{2}a_{1}a_{0}-a_{1}^{3}=0,\\ P^{4}\left(\eta \right):\; & 2\;\text{ln}\;{\left(\rho \right)}^{2}(6\zeta^{2}a_{2}a_{0}+\zeta^{2}a_{1}^{2}+5\zeta \nu a_{2}a_{1})-3a_{2}^{2}a_{0}-3a_{2}a_{1}^{2}=0,\\ P^{5}\left(\eta \right):\; & 4\;\text{ln}\;{\left(\rho \right)}^{2}(2\zeta^{2}a_{2}a_{1}+\zeta \nu a_{2}^{2})-3a_{2}^{2}a_{1}=0,\\ P^{6}\left(\eta \right):\; & 4\;\text{ln}\;{\left(\rho \right)}^{2}\zeta^{2}a_{2}^{2}-a_{2}^{3}=0. \end{array} \end{array}$$

The set of solutions below is produced by utilising a computational program to solve the aforementioned algebraic equations for the parameters *a*_0_, *a*_1_, *a*_2_, *μ*, *ν*, *q* and *ζ*:

$$\begin{array}{c} \begin{array}{c} a_{2}=4\;\text{ln}\;{\left(\rho \right)}^{2}\zeta^{2},\;\;\;\;\mu =\frac{1}{4}\frac{a_{0}}{\;\text{ln}\;{\left(\rho \right)}^{2}\zeta },\;\;\;\;\nu =\frac{1}{4}\frac{a_{1}}{\;\text{ln}\;{\left(\rho \right)}^{2}\zeta },\;\;\;q=0. \end{array} \end{array}$$ (13)

**Case 1.** If *ζ* ≠ 0 and Φ < 0, then

when the values of *a*_2_, *μ* and *ν* are entered via Eq (13) into Eq (12), the following results for Eq (6) are produced:

$$\begin{array}{c} U_{1}=4\;\text{ln}\;{\left(\rho \right)}^{2}\zeta^{2}{\left[-\frac{\nu }{2\zeta }+\frac{\sqrt{-\Phi }}{2\zeta }{\text{tan}}_{\rho }(\frac{\sqrt{-\Phi }}{2}\eta )\right]}^{2}, \end{array}$$

$$\begin{array}{c} U_{2}=4\;\text{ln}\;{\left(\rho \right)}^{2}\zeta^{2}{\left[-\frac{\nu }{2\zeta }-\frac{\sqrt{-\Phi }}{2\zeta }{\text{cot}}_{\rho }(\frac{\sqrt{-\Phi }}{2}\eta )\right]}^{2}, \end{array}$$

$$\begin{array}{c} U_{3}=4\;\text{ln}\;{\left(\rho \right)}^{2}\zeta^{2}{\left[-\frac{\nu }{2\zeta }+\frac{\sqrt{-\Phi }}{2\zeta }({\text{tan}}_{\rho }(\sqrt{-\Phi }\eta )\pm \sqrt{mn}\;{\text{sec}}_{\rho }(\sqrt{-\Phi }\eta ))\right]}^{2}, \end{array}$$

$$\begin{array}{c} U_{4}=4\;\text{ln}\;{\left(\rho \right)}^{2}\zeta^{2}{\left[-\frac{\nu }{2\zeta }+\frac{\sqrt{-\Phi }}{2\zeta }({\text{cot}}_{\rho }(\sqrt{-\Phi }\eta )\pm \sqrt{mn}\;{\text{csc}}_{\rho }(\sqrt{-\Phi }\eta ))\right]}^{2}, \end{array}$$

$$\begin{array}{c} U_{5}=4\;\text{ln}\;{\left(\rho \right)}^{2}\zeta^{2}{\left[-\frac{\nu }{2\zeta }+\frac{\sqrt{-\Phi }}{4\zeta }({\text{tan}}_{\rho }(\frac{\sqrt{-\Phi }}{4}\eta )-{\text{cot}}_{\rho }(\frac{\sqrt{-\Phi }}{4}\eta ))\right]}^{2}. \end{array}$$

**Case 2.** If *ζ* ≠ 0 and Φ > 0, then

$$\begin{array}{c} U_{6}=4\;\text{ln}\;{\left(\rho \right)}^{2}\zeta^{2}{\left[-\frac{\nu }{2\zeta }-\frac{\sqrt{\Phi }}{2\zeta }\;{\text{tanh}}_{\rho }(\frac{\sqrt{\Phi }}{2}\eta )\right]}^{2}, \end{array}$$

$$\begin{array}{c} U_{7}=4\;\text{ln}\;{\left(\rho \right)}^{2}\zeta^{2}{\left[-\frac{\nu }{2\zeta }-\frac{\sqrt{\Phi }}{2\zeta }{\text{coth}}_{\rho }(\frac{\sqrt{\Phi }}{2}\eta )\right]}^{2}, \end{array}$$

$$\begin{array}{c} U_{8}=4\;\text{ln}\;{\left(\rho \right)}^{2}\zeta^{2}{\left[-\frac{\nu }{2\zeta }+\frac{\sqrt{\Phi }}{2\zeta }(-{\text{tanh}}_{\rho }(\sqrt{\Phi }\eta )\pm i{\sqrt{mn}}_{\rho }(\sqrt{\Phi }\eta ))\right]}^{2}, \end{array}$$

$$\begin{array}{c} U_{9}=4\;\text{ln}\;{\left(\rho \right)}^{2}\zeta^{2}{\left[-\frac{\nu }{2\zeta }+\frac{\sqrt{\Phi }}{2\zeta }(-{\text{coth}}_{\rho }(\sqrt{\Phi }\eta )\pm {\sqrt{mn}}_{\rho }(\sqrt{\Phi }\eta ))\right]}^{2}, \end{array}$$

$$\begin{array}{c} U_{10}=4\;\text{ln}\;{\left(\rho \right)}^{2}\zeta^{2}{\left[-\frac{\nu }{2\zeta }-\frac{\sqrt{\Phi }}{4\zeta }({\text{tanh}}_{\rho }(\frac{\sqrt{\Phi }}{4}\eta )+{\text{coth}}_{\rho }(\frac{\sqrt{\Phi }}{4}\eta ))\right]}^{2}. \end{array}$$

**Case 3.** If *ν* = 0 and *μζ* > 0, then

$$\begin{array}{c} U_{11}=4\;\text{ln}\;{\left(\rho \right)}^{2}\zeta^{2}{\left[\sqrt{\frac{\mu }{\zeta }}\;{\text{tan}}_{\rho }(\sqrt{\mu \zeta }\eta )\right]}^{2}, \end{array}$$

$$\begin{array}{c} U_{12}=4\;\text{ln}\;{\left(\rho \right)}^{2}\zeta^{2}{\left[-\sqrt{\frac{\mu }{\zeta }}\;{\text{cot}}_{\rho }(\sqrt{\mu \zeta }\xi )\right]}^{2}, \end{array}$$

$$\begin{array}{c} U_{13}=4\;\text{ln}\;{\left(\rho \right)}^{2}\zeta^{2}{\left[\sqrt{\frac{\mu }{\zeta }}({\text{tan}}_{\rho }(2\sqrt{\mu \zeta }\eta )\pm \sqrt{mn}\;{\text{sec}}_{\rho }(2\sqrt{\mu \zeta }\eta ))\right]}^{2}, \end{array}$$

$$\begin{array}{c} U_{14}=4\;\text{ln}\;{\left(\rho \right)}^{2}\zeta^{2}{\left[\sqrt{\frac{\mu }{\zeta }}(-{\text{cot}}_{\rho }(2\sqrt{\mu \zeta }\eta )\pm \sqrt{mn}\;{\text{csc}}_{\rho }(2\sqrt{\mu \zeta }\eta ))\right]}^{2}, \end{array}$$

$$\begin{array}{c} U_{15}=4\;\text{ln}\;{\left(\rho \right)}^{2}\zeta^{2}{\left[\frac{1}{2}\sqrt{\frac{\mu }{\zeta }}({\text{tan}}_{\rho }(\frac{\sqrt{\mu \zeta }}{2}\eta )-{\text{cot}}_{\rho }(\frac{\sqrt{\mu \zeta }}{2}\eta ))\right]}^{2}. \end{array}$$

**Case 4.** If *ν* = 0 and *μζ* < 0, then

$$\begin{array}{c} U_{16}=4\;\text{ln}\;{\left(\rho \right)}^{2}\zeta^{2}{\left[-\sqrt{-\frac{\mu }{\zeta }}\;{\text{tanh}}_{\rho }(\sqrt{-\mu \zeta }\eta )\right]}^{2}, \end{array}$$

$$\begin{array}{c} U_{17}=4\;\text{ln}\;{\left(\rho \right)}^{2}\zeta^{2}{\left[-\sqrt{-\frac{\mu }{\zeta }}\;{\text{coth}}_{\rho }(\sqrt{-\mu \zeta }\eta )\right]}^{2}, \end{array}$$

$$\begin{array}{c} U_{18}=4\;\text{ln}\;{\left(\rho \right)}^{2}\zeta^{2}{\left[\sqrt{-\frac{\mu }{\zeta }}(-{\text{tanh}}_{\rho }(2\sqrt{-\mu \zeta }\eta )\pm i{\sqrt{mn}}_{\rho }(2\sqrt{-\mu \zeta }\eta ))\right]}^{2}, \end{array}$$

$$\begin{array}{c} U_{19}=4\;\text{ln}\;{\left(\rho \right)}^{2}\zeta^{2}{\left[\sqrt{-\frac{\mu }{\zeta }}(-{\text{coth}}_{\rho }(2\sqrt{-\mu \zeta }\eta )\pm {\sqrt{mn}}_{\rho }(2\sqrt{-\mu \zeta }\eta ))\right]}^{2}, \end{array}$$

$$\begin{array}{c} U_{20}=4\;\text{ln}\;{\left(\rho \right)}^{2}\zeta^{2}{\left[-\frac{1}{2}\sqrt{-\frac{\mu }{\zeta }}({\text{tanh}}_{\rho }(\frac{\sqrt{-\mu \zeta }}{2}\eta )+{\text{coth}}_{\rho }(\frac{\sqrt{-\mu \zeta }}{2}\eta ))\right]}^{2}. \end{array}$$

**Case 5.** If *μ* = *ζ* and *ν* = 0, then

$$\begin{array}{c} U_{21}=4\;\text{ln}\;{\left(\rho \right)}^{2}\zeta^{2}{\left[{\text{tan}}_{\rho }(\mu \eta )\right]}^{2}, \end{array}$$

$$\begin{array}{c} U_{22}=4\;\text{ln}\;{\left(\rho \right)}^{2}\zeta^{2}{\left[-{\text{cot}}_{\rho }(\mu \eta )\right]}^{2}, \end{array}$$

$$\begin{array}{c} U_{23}=4\;\text{ln}\;{\left(\rho \right)}^{2}\zeta^{2}{\left[{\text{tan}}_{\rho }(2\mu \eta )\pm \sqrt{mn}\;{\text{sec}}_{\rho }(2\mu \eta )\right]}^{2}, \end{array}$$

$$\begin{array}{c} U_{24}=4\;\text{ln}\;{\left(\rho \right)}^{2}\zeta^{2}{\left[-{\text{cot}}_{\rho }(2\mu \eta )\pm \sqrt{mn}\;{\text{csc}}_{\rho }(2\mu \eta )\right]}^{2}, \end{array}$$

$$\begin{array}{c} U_{25}=4\;\text{ln}\;{\left(\rho \right)}^{2}\zeta^{2}{\left[\frac{1}{2}({\text{tan}}_{\rho }(\frac{\mu }{2}\eta )-{\text{cot}}_{\rho }(\frac{\mu }{2}\eta ))\right]}^{2}. \end{array}$$

**Case 6.** If *ζ* = −*μ* and *ν* = 0, then

$$\begin{array}{c} U_{26}=4\;\text{ln}\;{\left(\rho \right)}^{2}\mu^{2}{\left[-{\text{tanh}}_{\rho }(\mu \eta )\right]}^{2}, \end{array}$$

$$\begin{array}{c} U_{27}=4\;\text{ln}\;{\left(\rho \right)}^{2}\mu^{2}{\left[-{\text{coth}}_{\rho }(\mu \eta )\right]}^{2}, \end{array}$$

$$\begin{array}{c} U_{28}=4\;\text{ln}\;{\left(\rho \right)}^{2}\mu^{2}{\left[-{\text{tanh}}_{\rho }(2\mu \eta )\pm i{\sqrt{mn}}_{\rho }(2\mu \eta )\right]}^{2}, \end{array}$$

$$\begin{array}{c} U_{29}=4\;\text{ln}\;{\left(\rho \right)}^{2}\mu^{2}{\left[-{\text{cot}}_{\rho }(2\mu \eta )\pm {\sqrt{mn}}_{\rho }(2\mu \eta )\right]}^{2}, \end{array}$$

$$\begin{array}{c} U_{30}=4\;\text{ln}\;{\left(\rho \right)}^{2}\mu^{2}{\left[-\frac{1}{2}({\text{tanh}}_{\rho }(\frac{\mu }{2}\eta )+{\text{coth}}_{\rho }(\frac{\mu }{2}\eta ))\right]}^{2}. \end{array}$$

**Case 7.** If *ν* ≠ 0 and *μ* = 0, then

$$\begin{array}{c} U_{31}=4\;\text{ln}\;{\left(\rho \right)}^{2}\zeta^{2}{\left[-\frac{m\nu }{\zeta ({\text{cosh}}_{\rho }\left(\nu \eta \right)-{\text{sinh}}_{\rho }\left(\nu \eta \right)+m)}\right]}^{2}, \end{array}$$

$$\begin{array}{c} U_{32}=4\;\text{ln}\;{\left(\rho \right)}^{2}\zeta^{2}{\left[-\frac{\nu ({\text{sinh}}_{\rho }(\nu \eta )+{\text{cosh}}_{\rho }(\nu \eta ))}{\zeta ({\text{cosh}}_{\rho }(\nu \eta )+{\text{sinh}}_{\rho }(\nu \eta )+n)}\right]}^{2}. \end{array}$$

**Case 8.** If *ν* = *p*, *ζ* = *pq*, (*μ* = 0 and *q* ≠ 0), then

$$\begin{array}{c} U_{33}=4\;\text{ln}\;{\left(\rho \right)}^{2}{\left(pq\right)}^{2}{\left[-\frac{m\rho^{p\eta }}{m-qn\rho^{p\eta }}\right]}^{2}. \end{array}$$

**Case 9.** If *ν*^2^ = 4*μζ*, then

$$\begin{array}{c} U_{34}=4\;\text{ln}\;{\left(\rho \right)}^{2}\zeta^{2}{\left[\frac{-2\mu (\nu \eta \;\text{ln}\;\rho +2)}{\nu^{2}\eta \;\text{ln}\;\rho }\right]}^{2}. \end{array}$$

**Case 10.** If *μ* = *ν* = 0, then

$$\begin{array}{c} U_{35}=4\;\text{ln}\;{\left(\rho \right)}^{2}\zeta^{2}{\left[\frac{-1}{\zeta \eta \;\text{ln}\;\rho }\right]}^{2}. \end{array}$$

### Solutions by employing the modified auxiliary equation method

In this section, the generalized *q* deformed Sinh-Gordon equation will be solved by using the modified auxiliary equation method. Consider Eq (9), which takes the following as its solution:

$$\begin{array}{c} U\left(\eta \right)=a_{0}+\sum_{i=1}^{n}[a_{i}{\left(k^{g}\right)}^{i}+b_{i}{\left(k^{g}\right)}^{-i})], \end{array}$$ (14)

where *g*(*η*) fits the auxiliary equation below and *a*_*i*_ and *b*_*i*_ are constants that must be computed. While

$$\begin{array}{c} g^{\prime }\left(\eta \right)=\frac{\beta +\theta k^{-g}+\sigma k^{g}}{\;\text{ln}\;k}, \end{array}$$ (15)

where *σ*, *β*, *θ* and *k* are undefined constants with *k* > 0, *k* ≠ 1. By balancing the nonlinear highest terms with highest derivatives into Eq (6), the value of *n* = 2 is obtained. The Eq (14) is convert in the following form as by using the above information:

$$\begin{array}{c} U\left(\eta \right)=a_{0}+a_{1}k^{g}+a_{2}k^{2g}+b_{1}k^{-g}+b_{2}k^{-2g}. \end{array}$$ (16)

After employing Eq (15) with Eq (16) into Eq (6), set of algebraic equations turns up as follows and setting all the factors of the distinct powers of *k*^*g*^ equal to zero, we have

$$\begin{array}{c} \begin{array}{cc} {\left(K^{g(\eta )}\right)}^{0}:\; & 2a_{0}a_{1}\theta \beta +4a_{0}a_{2}\theta^{2}+2a_{0}b_{1}\beta \sigma +4a_{0}b_{2}\sigma^{2}-2a_{1}^{2}\theta^{2}+16a_{1}b_{1}\theta \sigma +8a_{1}b_{1}\beta^{2}\\ & +34a_{1}b_{2}\beta \sigma +34a_{2}b_{1}\theta \beta +64a_{2}b_{2}\theta \sigma +32a_{2}b_{2}\beta^{2}-2b_{1}^{2}\sigma^{2}-a_{0}^{3}-6a_{0}a_{1}b_{1}\\ & -6a_{0}a_{2}b_{2}-3a_{1}^{2}b_{2}-3a_{2}b_{1}^{2}+a_{0}q=0,\\ {\left(K^{g(\eta )}\right)}^{1}:\; & 4a_{0}a_{1}\theta \sigma +2a_{0}a_{1}\beta^{2}+12a_{0}a_{2}\theta \beta -2a_{1}^{2}\theta \beta -4a_{1}a_{2}\theta^{2}+16a_{1}b_{1}\beta \sigma +16a_{1}b_{2}\sigma^{2}\\ & +36a_{2}b_{1}\theta \sigma +18a_{2}b_{1}\beta^{2}+64a_{2}b_{2}\beta \sigma -3a_{0}^{2}a_{1}-6a_{0}a_{2}b_{1}-3a_{1}^{2}b_{1}-6a_{1}a_{2}b_{2}\\ & +a_{1}q=0,\\ {\left(K^{g(\eta )}\right)}^{2}:\; & 6a_{0}a_{1}\beta \sigma +16a_{0}a_{2}\theta \sigma +8a_{0}a_{2}\beta^{2}-2a_{1}a_{2}\theta \beta +8a_{1}b_{1}\sigma^{2}-4a_{2}^{2}\theta^{2}+38a_{2}b_{1}\beta \sigma \\ & +32a_{2}b_{2}\sigma^{2}-3a_{0}^{2}a_{2}-3a_{0}a_{1}^{2}-6a_{1}a_{2}b_{1}-3a_{2}^{2}b_{2}+a_{2}q=0,\\ {\left(K^{g(\eta )}\right)}^{3}:\; & 4a_{0}a_{1}\sigma^{2}+20a_{0}a_{2}\beta \sigma +2a_{1}^{2}\beta \sigma +4a_{1}a_{2}\sigma \theta +2a_{1}a_{2}\beta^{2}-4a_{2}^{2}\theta \beta +20a_{2}b_{1}\sigma^{2}\\ & -6a_{0}a_{1}a_{2}-a_{1}^{3}-3a_{2}^{2}b_{1}=0,\\ {\left(K^{g(\eta )}\right)}^{4}:\; & 3a_{0}(4a_{2}\sigma^{2}-a_{2}^{2})+a_{1}^{2}(2\sigma^{2}-3a_{2})+10a_{1}a_{2}\beta \sigma =0,\\ {\left(K^{g(\eta )}\right)}^{5}:\; & 8a_{1}a_{2}\sigma^{2}+a_{2}^{2}(4\beta \sigma -3a_{1})=0,\\ {\left(K^{g(\eta )}\right)}^{6}:\; & 4a_{2}^{2}\sigma^{2}-a_{2}^{3}=0,\\ \frac{1}{{\left(K^{g(\eta )}\right)}^{1}}:\; & 4a_{0}b_{1}\theta \sigma +2a_{0}b_{1}\beta^{2}+12a_{0}b_{2}\beta \sigma +16a_{1}b_{1}\theta \beta +36a_{1}b_{2}\theta \sigma +18a_{1}b_{2}\beta^{2}\\ & +16a_{2}b_{1}\theta^{2}+64a_{2}b_{2}\theta \beta -2b_{1}^{2}\beta \sigma -4b_{1}b_{2}\sigma^{2}-3a_{0}^{2}b_{1}-6a_{0}a_{1}b_{2}-3a_{1}b_{1}^{2}\\ & -6a_{2}b_{1}b_{2}+b_{1}q=0,\\ \frac{1}{{\left(K^{g(\eta )}\right)}^{2}}:\; & 6a_{0}b_{1}\theta \beta +16a_{0}b_{2}\theta \sigma +8a_{0}b_{2}\beta^{2}+8a_{1}b_{1}\theta^{2}+38a_{1}b_{2}\theta \beta +32a_{2}b_{2}\theta^{2}-2b_{1}b_{2}\beta \sigma \\ & -4b_{2}^{2}\sigma^{2}-3a_{0}^{2}b_{2}-3a_{0}b_{1}^{2}-6a_{1}b_{1}b_{2}-3a_{2}b_{2}^{2}+b_{2}q=0,\\ \frac{1}{{\left(K^{g(\eta )}\right)}^{3}}:\; & 4a_{0}b_{1}\theta^{2}+20a_{0}b_{2}\theta \sigma +20a_{1}b_{2}\theta^{2}+2b_{1}^{2}\theta \beta +4b_{1}b_{2}\theta +21b_{2}\beta^{2}-4b_{2}^{2}\beta \sigma -6a_{0}b_{1}b_{2}\\ & -3a_{1}b_{2}^{2}-b_{1}^{3}=0,\\ \frac{1}{{\left(K^{g(\eta )}\right)}^{4}}:\; & 3a_{0}(4b_{2}\theta^{2}-b_{2}^{2})+b_{1}^{2}(2\theta^{2}-3b_{2})+10\theta \beta b_{1}b_{2}=0,\\ \frac{1}{{\left(K^{g(\eta )}\right)}^{5}}:\; & 8\theta^{2}b_{1}b_{2}+b_{2}^{2}(4\theta \beta -3b_{1})=0,\\ \frac{1}{{\left(K^{g(\eta )}\right)}^{6}}:\; & 4b_{2}^{2}\theta^{2}-b_{2}^{3}=0. \end{array} \end{array}$$

The set of solutions below is produced by utilising a computational program to solve the aforementioned algebraic equations for the parameters *a*_0_, *a*_1_, *a*_2_, *b*_1_, *b*_2_, *β* and *σ*:

$$\begin{array}{c} \begin{array}{c} a_{0}=\pm \frac{1}{2}\sqrt{q},\;\;\;a_{1}=0,\;\;a_{2}=\frac{1}{64}\frac{q}{\theta^{2}},\;\;\;b_{1}=0,\;\;b_{2}=4\theta^{2},\;\;\sigma =\pm \frac{1}{16}\frac{\sqrt{q}}{\theta }. \end{array} \end{array}$$ (17)

Plugging the values *a*_0_, *a*_1_, *a*_2_, *b*_1_,*b*_2_, *σ*, and *β* via Eq (17) into Eq (16), it will turns up the examining solutions for the Eq (6):

For *β*^2^ − 4*σθ* < 0 and *σ* ≠ 0 shows,

$$\begin{array}{c} \begin{array}{cc} m_{1}= & \pm \frac{1}{2}\sqrt{q}+\frac{1}{64}\frac{q}{\theta^{2}}{\left[\frac{-\beta +\sqrt{4\sigma \theta -\beta^{2}}\text{tan}(\frac{\sqrt{4\sigma \theta -\beta^{2}}\eta }{2})}{2\sigma }\right]}^{2}\\ & +4\theta^{2}{\left[\frac{-\beta +\sqrt{4\sigma \theta -\beta^{2}}\text{tan}(\frac{\sqrt{4\sigma \theta -\beta^{2}}\eta }{2})}{2\sigma }\right]}^{-2}, \end{array} \end{array}$$ (18)

or

$$\begin{array}{c} \begin{array}{cc} m_{2}= & \pm \frac{1}{2}\sqrt{q}+\frac{1}{64}\frac{q}{\theta^{2}}{\left[-\frac{\beta +\sqrt{4\sigma \theta -\beta^{2}}\text{cot}(\frac{\sqrt{4\sigma \theta -\beta^{2}}\eta }{2})}{2\sigma }\right]}^{2}\\ & +4\theta^{2}{\left[-\frac{\beta +\sqrt{4\sigma \theta -\beta^{2}}\text{cot}(\frac{\sqrt{4\sigma \theta -\beta^{2}}\eta }{2})}{2\sigma }\right]}^{-2}. \end{array} \end{array}$$ (19)

For *β*^2^ − 4*σθ* > 0 and *σ* ≠ 0 shows,

$$\begin{array}{c} \begin{array}{cc} m_{3}= & \pm \frac{1}{2}\sqrt{q}+\frac{1}{64}\frac{q}{\theta^{2}}{\left[-\frac{\beta +\sqrt{\beta^{2}-4\sigma \theta }\;\text{tanh}(\frac{\sqrt{\beta^{2}-4\sigma \theta }\eta }{2})}{2\sigma }\right]}^{2}\\ & +4\theta^{2}{\left[-\frac{\beta +\sqrt{\beta^{2}-4\sigma \theta }\;\text{tanh}(\frac{\sqrt{\beta^{2}-4\sigma \theta }\eta }{2})}{2\sigma }\right]}^{-2}, \end{array} \end{array}$$ (20)

or

$$\begin{array}{c} \begin{array}{cc} m_{4}= & \pm \frac{1}{2}\sqrt{q}+\frac{1}{64}\frac{q}{\theta^{2}}{\left[-\frac{\beta +\sqrt{\beta^{2}-4\sigma \theta }\;\text{coth}(\frac{\sqrt{\beta^{2}-4\sigma \theta }\eta }{2})}{2\sigma }\right]}^{2}\\ & +4\theta^{2}{\left[-\frac{\beta +\sqrt{\beta^{2}-4\sigma \theta }\;\text{coth}(\frac{\sqrt{\beta^{2}-4\sigma \theta }\eta }{2})}{2\sigma }\right]}^{-2}. \end{array} \end{array}$$ (21)

For *β*^2^ − 4*σθ* = 0 and *σ* ≠ 0 shows,

$$\begin{array}{c} m_{5}=\pm \frac{1}{2}\sqrt{q}+\frac{1}{64}\frac{q}{\theta^{2}}{\left[-\frac{2+\beta \eta }{2\sigma \eta }\right]}^{2}+4\theta^{2}{\left[-\frac{2+\beta \eta }{2\sigma \eta }\right]}^{-2}. \end{array}$$ (22)

## Results and discussions

**Fig 1:**
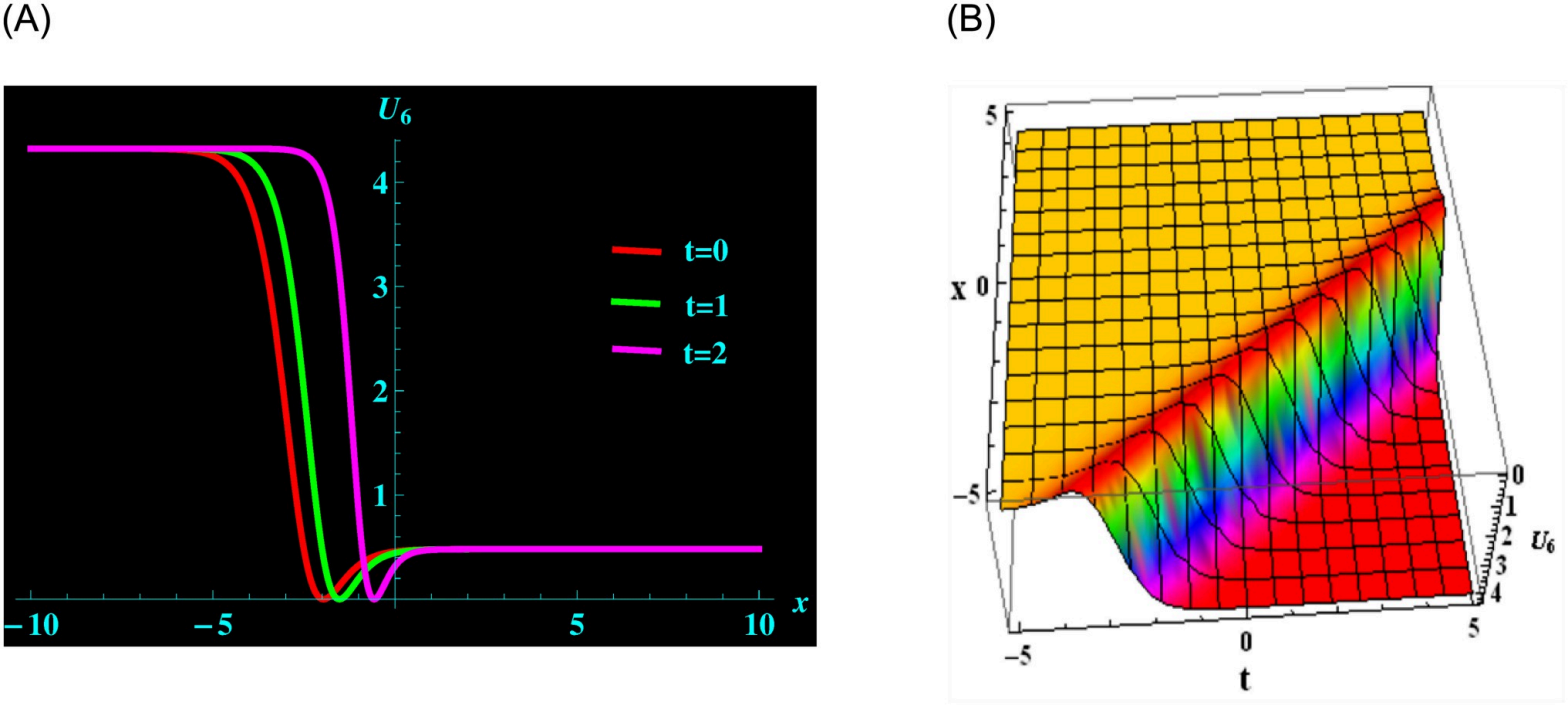
Graphical depiction of kink-bright soliton shapes for *U*_6_ in 2D (the red line corresponds to t = 0, the green line to t = 1 and the magenta line to t = 2) and 3D sketches. (a) 2D portrait for *U*_6_. (b) 3D portrait for *U*_6_.

The chosen outcomes to the wave equation are displayed graphically in this section in 2D and 3D forms. The wave solution has been used to construct the many types of graphs. The structure of the travelling wave varies along with the unexplained variables of the outcomes. The composition of the solution has been examined. Now, the graphs are shown illustrating how the generalized *q* deformed Sinh-Gordon equation has changed over time. There are several different solution of the generalized *q* deformed Sinh-Gordon equation that contain unspecified parameters. These unknown factors have been effect on the nature of the solutions. In other words, if different specific values are assigned to the variables, several types of solutions can be produced from the general solution. The following diagram illustrates how the solution *U*_6_ is affected by the parameters:

The variables *ρ*, *ζ*, *μ*, *ν*, *y*, and *α* are all included in the inclusive finding *U*_6_. For the following values: *ρ* = 2, *ζ* = 1.5, *μ* = −0.5, *ν* = −1, *y* = 2.5, and *α* = 0.2 from solution *U*_6_, we acquire the kink-bright soliton structure. A 2D graph for oscillating with temporal components *t* = 0, 1 and 2 and components of velocity *α* = 0.2, 0.5, 0.8 within the limit −10 ≤ *x* ≤ 10 is shown in (Fig 1). Additionally, at intervals −5 ≤ *x* ≤ 5 and −5 ≤ *t* ≤ 5 and 3D graphs are displayed. Additionally, at intervals −5 ≤ *x* ≤ 5 and −5 ≤ *t* ≤ 5 and 3D graphs are displayed.

**Fig 2:**
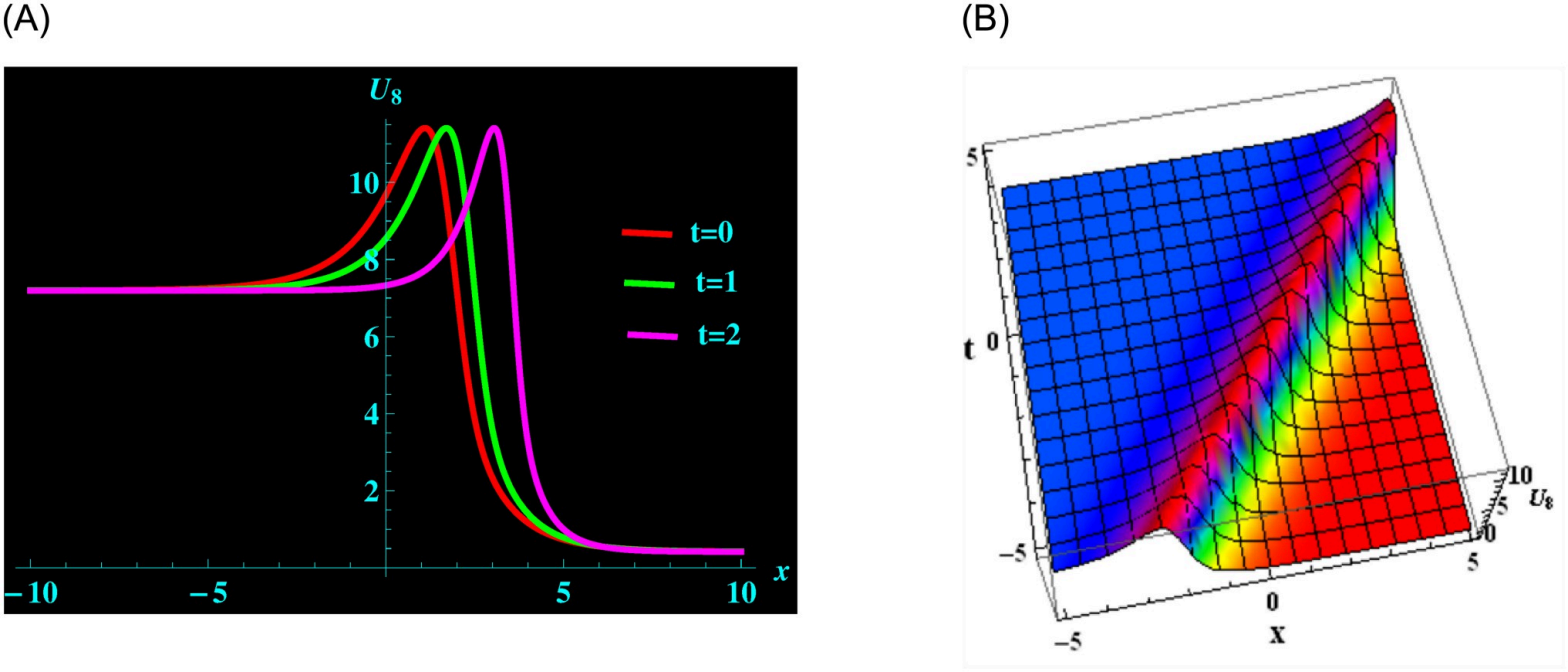
Graphical depiction of bright soliton shapes for *U*_8_ in 2D (the red line corresponds to t = 0, the green line to t = 1 and the magenta line to t = 2) and 3D sketches. (a) 2D portrait for *U*_8_. (b) 3D portrait for *U*_8_.

The specific solution *U*_8_ is made up of the parameters *ρ*, *ζ*, *μ*, *ν*, *y* and *α*. For the following values: *ρ* = 2, *ζ* = −1.8, *μ* = −0.5, *ν* = −2.4, *y* = −2 and *α* = 0.2 from solution *U*_8_, we are able to obtain the bright soliton structure. (Fig 2) shows a 2D graph for oscillating with temporal components *t* = 0, 1 and 2 and components of velocity *α* = 0.2, 0.5, 0.8 within the limit −10 ≤ *x* ≤ 10. In addition, at intervals −5 ≤ *x* ≤ 5 and −5 ≤ *t* ≤ 5 and 3D graphs are exhibited.

**Fig 3:**
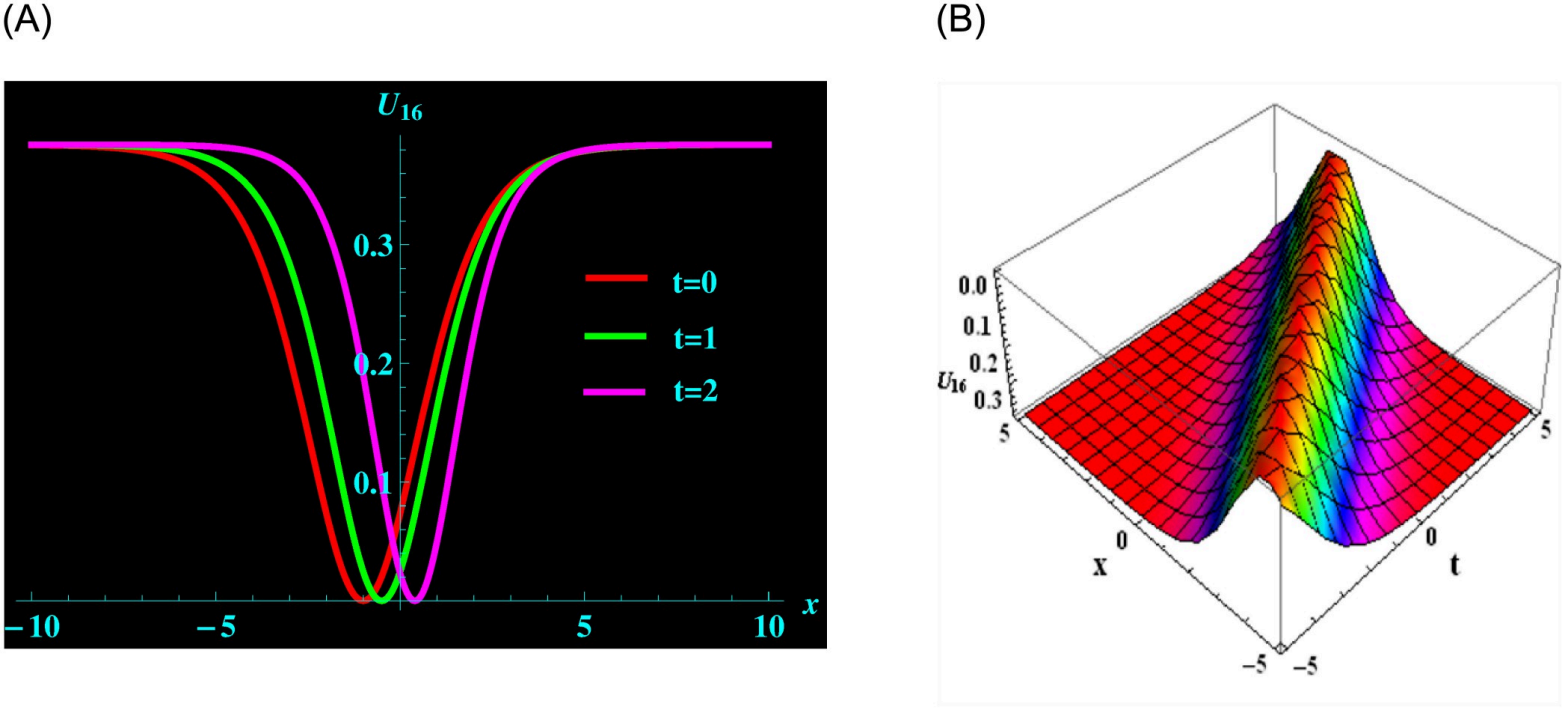
Graphical depiction of bright soliton shapes for *U*_16_ in 2D (the red line corresponds to t = 0, the green line to t = 1 and the magenta line to t = 2) and 3D sketches. (a) 2D portrait for *U*_16_. (b) 3D portrait for *U*_16_.

The specific solution *U*_16_ is made up of the parameters *ρ*, *ζ*, *μ*, *y* and *α*. For the following values: *ρ* = 2, *ζ* = 0.2, *μ* = −1, *y* = 1 and *α* = 0.3 from solution *U*_16_, we achieve dark bright soliton structure. (Fig 3) depicts a 2D graph for oscillating with temporal components *t* = 0, 1 and 2 and components of velocity *α* = 0.3, 0.5, 0.7 within the limit −10 ≤ *x* ≤ 10. Moreover, at intervals −5 ≤ *x* ≤ 5 and −5 ≤ *t* ≤ 5 and 3D graphs are presented.

**Fig 4:**
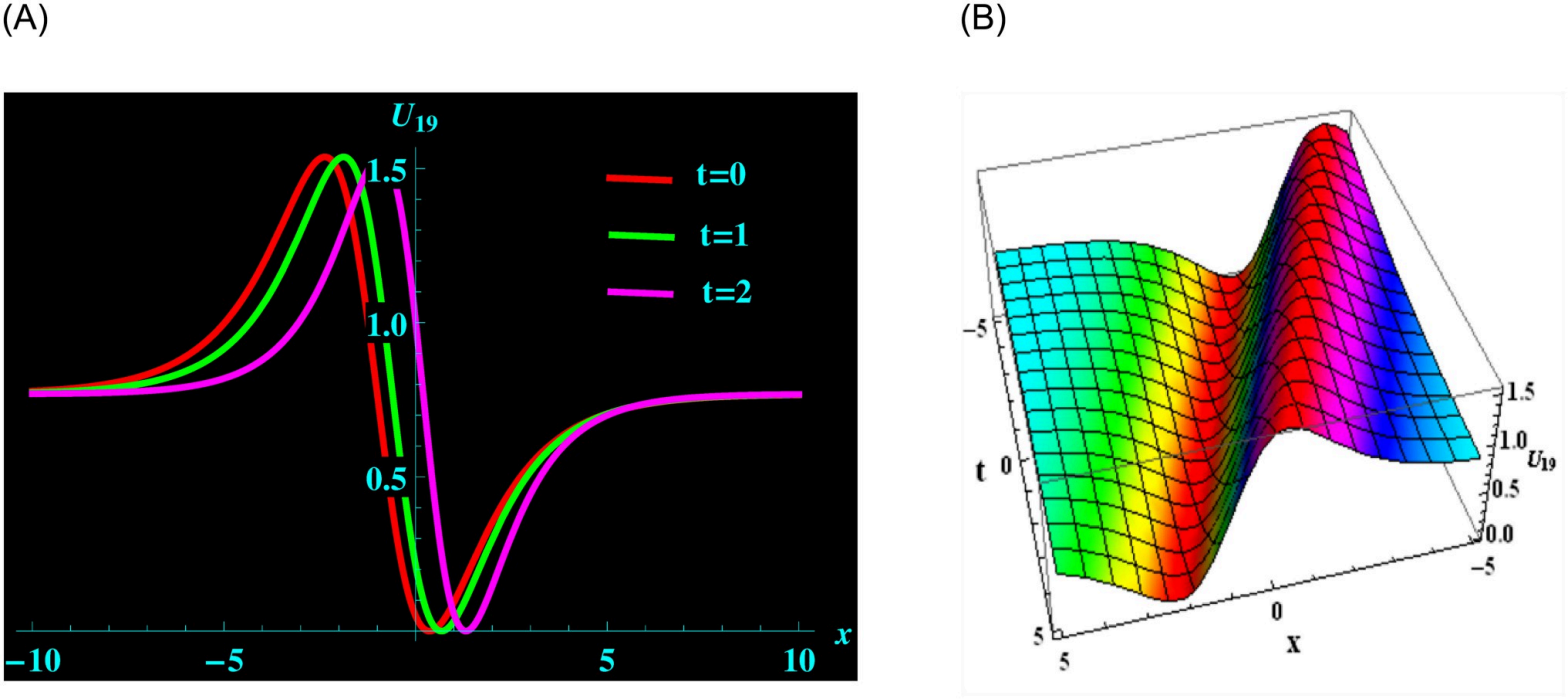
Graphical depiction of bright-dark soliton shapes for *U*_19_ in 2D (the red line corresponds to t = 0, the green line to t = 1 and the magenta line to t = 2) and 3D sketches. (a) 2D portrait for *U*_19_. (b) 3D portrait for *U*_19_.

The distinct solution *U*_19_ is made up of the parameters *ρ*, *ζ*, *μ*, *y*, and *α*. For the following values: *ρ* = 4, *ζ* = 0.2, *μ* = −0.5, *y* = 1, and *α* = 0.2 from solution *U*_19_, we get bright-dark soliton structure. (Fig 4) portrays a 2D graph for oscillating with temporal components *t* = 0, 1 and 2 and components of velocity *α* = 0.3, 0.4, 0.6 within the limit −10 ≤ *x* ≤ 10. Furthermore, at intervals −5 ≤ *x* ≤ 5 and −5 ≤ *t* ≤ 5 and 3D graphs are demonstrated.

**Fig 5:**
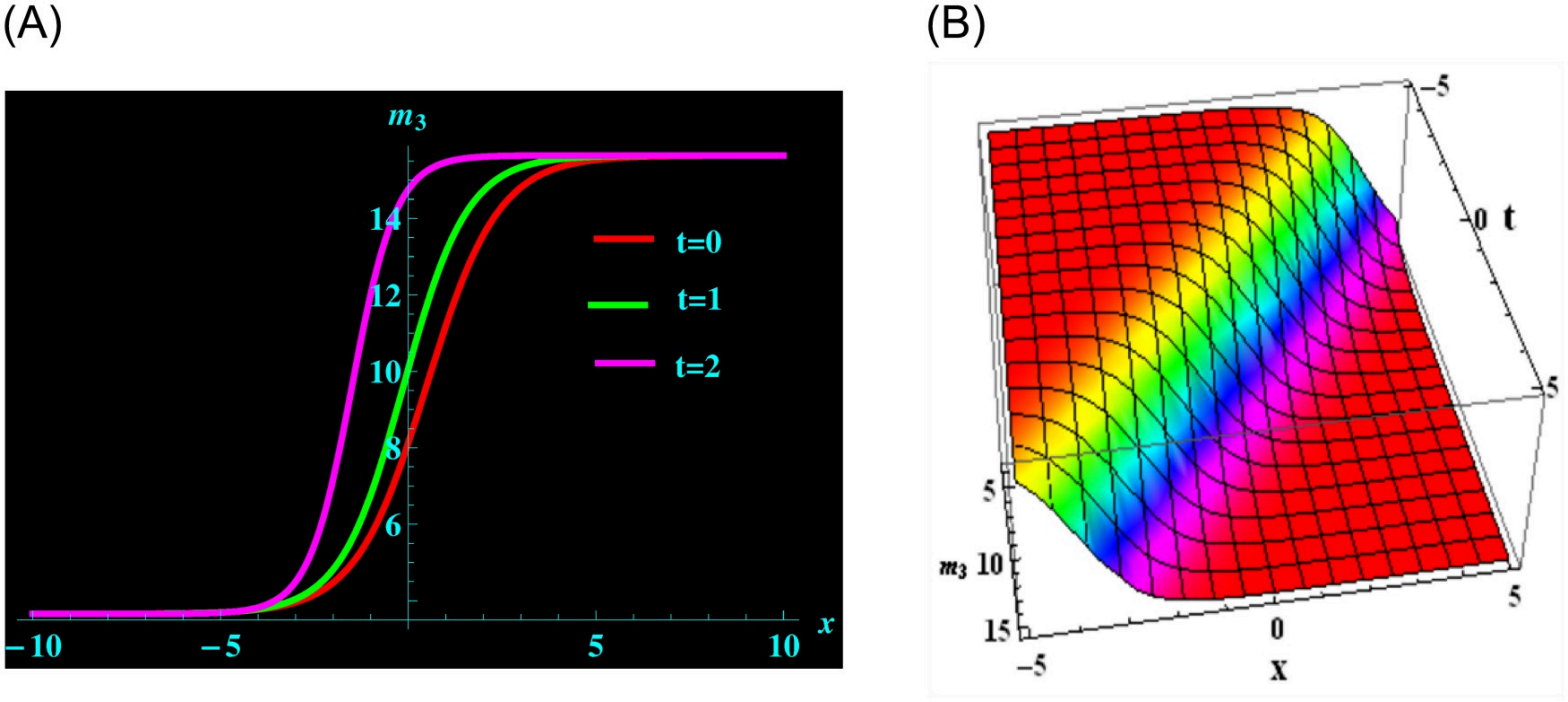
Graphical depiction of kink soliton shapes for *m*_3_ in 2D (the red line corresponds to t = 0, the green line to t = 1 and the magenta line to t = 2) and 3D sketches. (a) 2D portrait for *m*_3_. (b) 3D portrait for *m*_3_.

The particular solution *m*_3_ is formed up of the parameters *q*, *θ*, *σ*, *y*, *β* and *α*. For the following values: *q* = 0.5, *θ* = 2, *σ* = 1, *y* = 0.5, *β* = −3 and *α* = −0.2 from solution *m*_3_, we derive kink soliton structure. (Fig 5) represents a 2D graph for oscillating with temporal components *t* = 0, 1 and 2 and components of velocity *α* = −0.2, −0.5, −0.8 within the limit −10 ≤ *x* ≤ 10. Further, at intervals −5 ≤ *x* ≤ 5 and −5 ≤ *t* ≤ 5 and 3D graphs are revealed.

**Fig 6:**
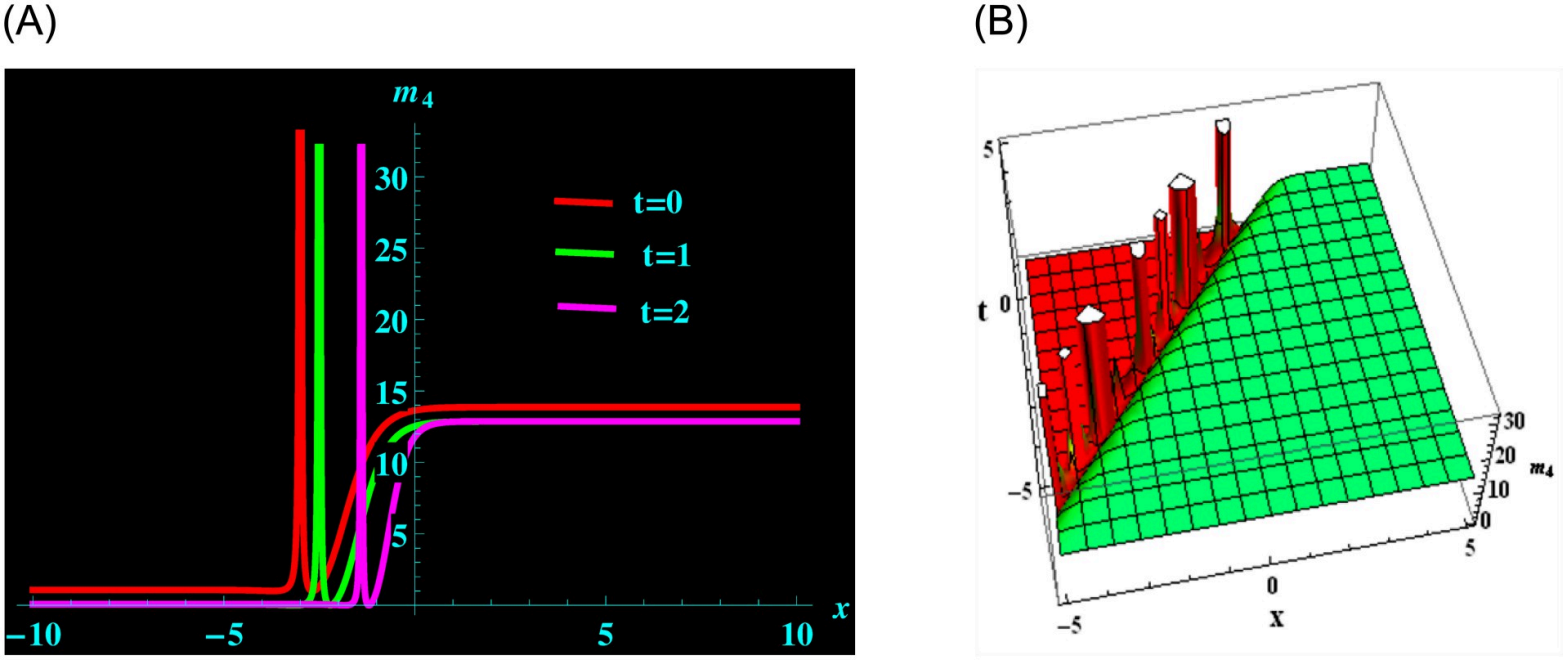
Graphical depiction of kink-peakon soliton shapes for *m*_4_ in 2D (the red line corresponds to t = 0, the green line to t = 1 and the magenta line to t = 2) and 3D sketches. (a) 3D portrait for *m*_4_. (b) 3D portrait for *m*_4_.

The particular result *m*_4_ is formed up of the parameters *q*, *θ*, *σ*, *y*, *β* and *α*. For the following values: *q* = 1, *θ* = 0.3, *σ* = −2, *y* = 3, *β* = −1.5 and *α* = 0.2 from solution *m*_4_, we derive kink-peakon soliton structure. (Fig 6) demonstrates a 2D graph for oscillating with temporal components *t* = 0, 1 and 2 and components of velocity *α* = 0.2, 0.5, 0.8 within the limit −10 ≤ *x* ≤ 10. Further, at intervals −5 ≤ *x* ≤ 5 and −5 ≤ *t* ≤ 5 and 3D graphs are showed.

## Investigation of chaotic behavior in perturbed dynamical system

**Fig 7:**
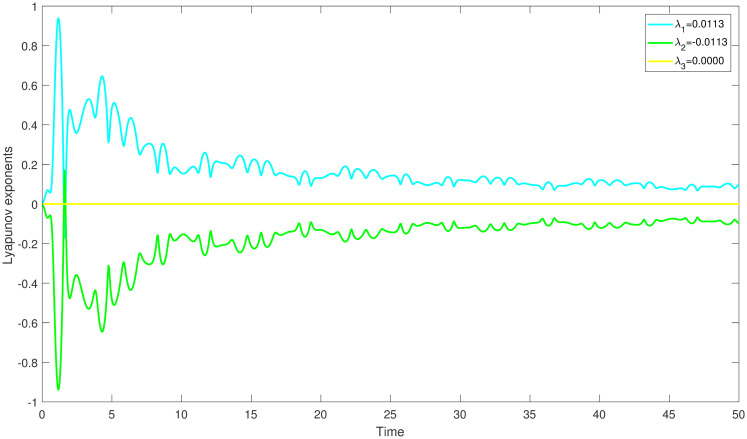
The study of chaotic behaviour for system (23) through Lyapunov exponents is conducted with parameters *K*_1_ = 3, *K*_2_ = −3.5, *K*_3_ = 5.2, Ω = 3.7, *θ* = 1.8 and the starting conditions (0.6,0.6,0.6).

**Fig 8:**
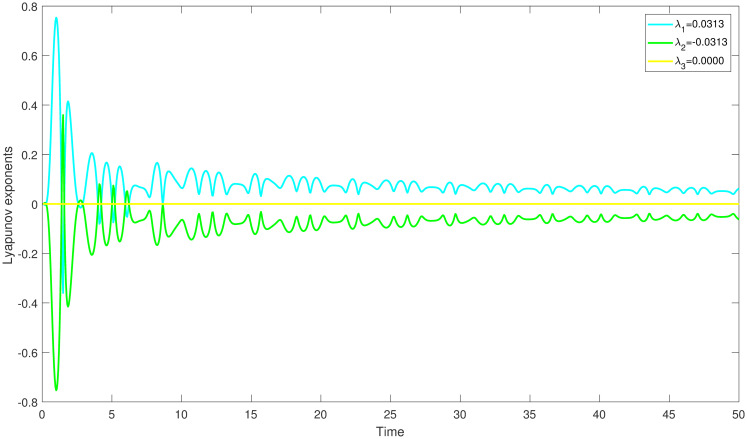
The study of chaotic behaviour for system (23) through Lyapunov exponents is conducted with parameters *K*_1_ = 3, *K*_2_ = −3.5, *K*_3_ = 5.2, Ω = 3.7, *θ* = 1.8 and the starting conditions (0.4,0.4,0.4).

**Fig 9:**
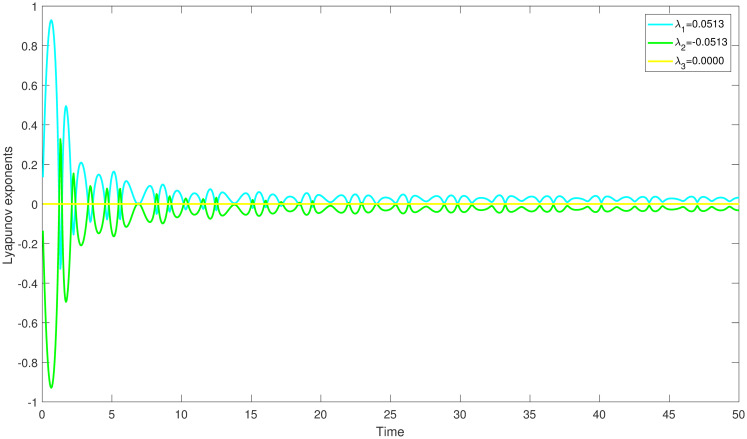
The study of chaotic behaviour for system (23) through Lyapunov exponents is conducted with parameters *K*_1_ = 3, *K*_2_ = −3.5, *K*_3_ = 5.2, Ω = 3.7, *θ* = 1.8 and the starting conditions (0.1,0.1,0.1).

This segment delves at the analysis of Eq (6), which describes quasi-periodic and chaotic behaviour. Employing a Galilean transformation, the studied equation is transformed into a planar dynamical system. To examine the chaotic behaviour of the planar dynamical system, a perturbation phrase Ω cos(*θη*) is introduced. Consequently, the dynamical planar system and the perturbation phrase have the following structure:

$$\begin{array}{c} \left\{ \begin{array}{c} \frac{dm}{d\eta }=N,\\ \frac{dN}{d\eta }=K_{1}m^{2}+K_{2}m-K_{3}+\Omega \;\text{cos}\left(W\right),\\ \frac{dW}{d\eta }=\theta , \end{array}\right. \end{array}$$ (23)

with *W* = *θη*, is an independent system. The aforementioned system creates a disturbance phrase which represent the force and its frequency using the parameters Ω and *θ*. Behaviour of a system may change and looks chaotic whenever it is impacted by outside forces. In our research, we discover this behaviour in a chaotically behaving system (23) which demonstrate unpredictable time dependent trajectories that diverge from predictable trends. For determining chaos, we applied Lyapunov exponents method, multistability and time series analysis and bifurcation diagram. In order to comprehend the perturbed dynamical system, we then displayed the behaviour of the aforementioned exponents over time. We can figure out the chaotic behaviour of perturbed dynamical system at *K*_1_ = 3, *K*_2_ = −3.5, *K*_3_ = 5.2, Ω = 3.7, *θ* = 1.8 and the starting conditions (0.6,0.6,0.6), (0.4,0.4,0.4) and (0.1,0.1,0.1) correspondingly by displaying the resulting Lyapunov exponents versus change in time in Figs (7–9).

In (Fig 7), we have observed that the system (23) is chaotic at λ_1_ = 0.0113, which is the largest positive Lyapunov exponent. This clearly shows that the orbits are diverging. In the same way, the largest positive Lyapunov exponent, λ_1_ = 0.0313 for the perturbed dynamical system (23) is represents the presence of chaos. Moreover, the largest positive Lyapunov exponent, λ_1_ = 0.0513 for the perturbed dynamical system (23) is represents the presence of chaos.

**Fig 10:**
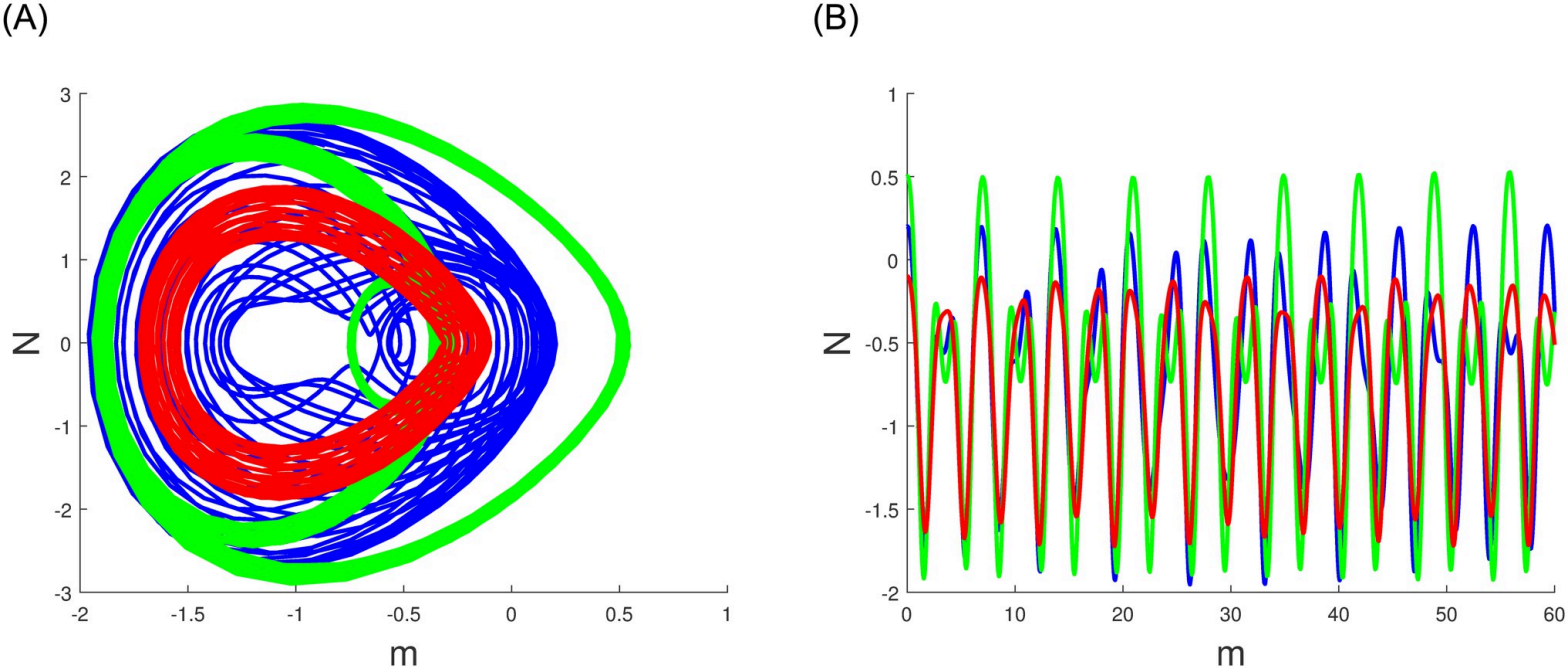
The study of chaotic behavior for system (23) through multistability and time series analysis under diverse beginning conditions: Blue (0.2,0.05,0), green (0.5,0.1,0) and red (-0.1,0.02,0). (a) Multistability analysis. (b) Time series analysis.

**Fig 11:**
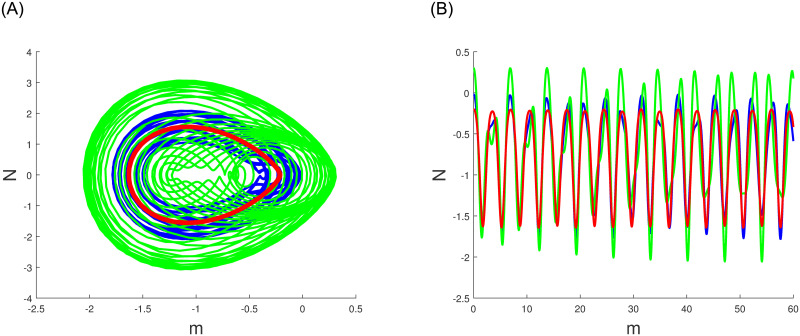
The study of chaotic behavior for system (23) through multistability and time series analysis under diverse beginning conditions: Blue (-0.02,0.01,0), green (0.3,-0.01,0) and red (-0.2,-0.01,0). (a) Multistability analysis. (b) Time series analysis.

**Fig 12:**
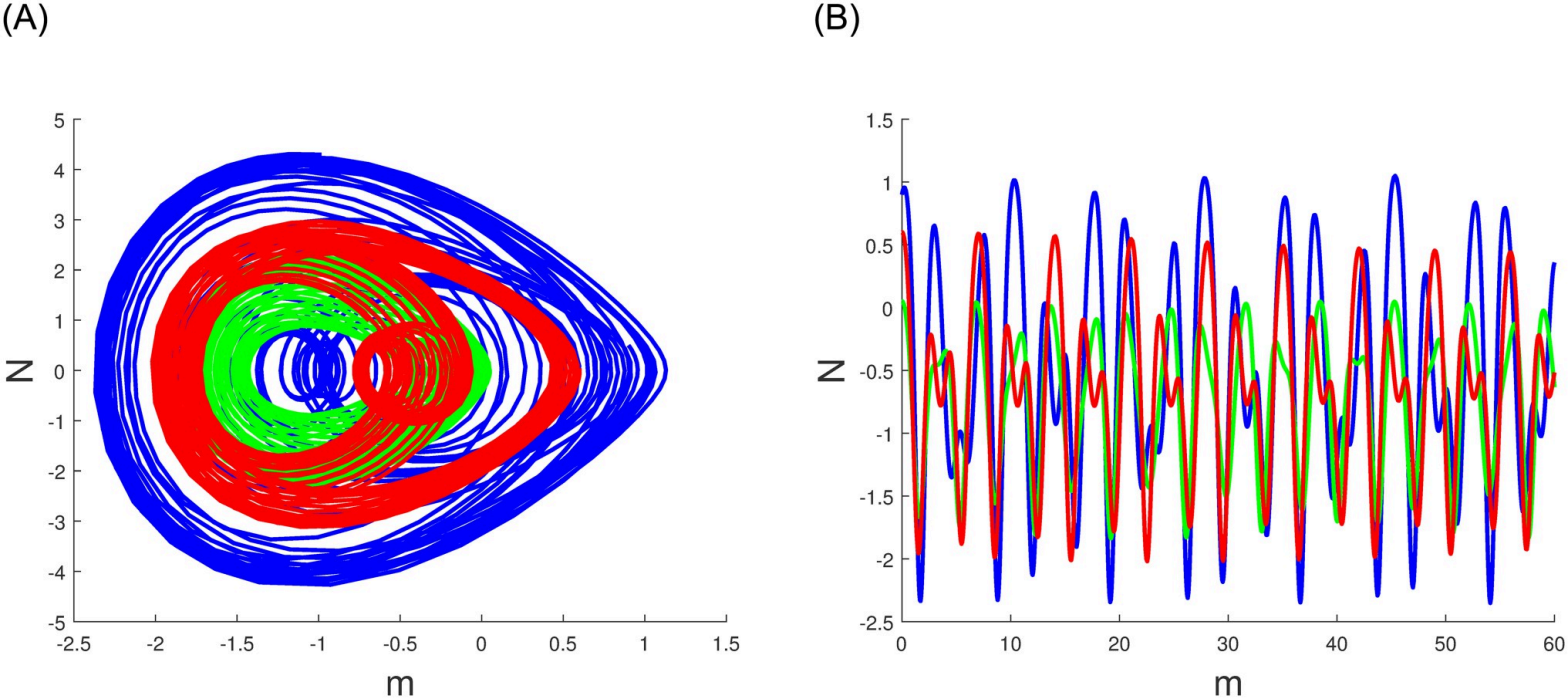
The study of chaotic behavior for system (23) through multistability and time series analysis under diverse beginning conditions: Blue (0.9,0.5,0), green (0.05,0.02,0) and red (0.6,0.04,0). (a) Multistability analysis. (b) Time series analysis.

A characteristic of perturbed dynamical systems known as multistability and time series analysis denotes the presence of multiple alternative dynamic behaviors with an identical parameters set but diverse primary conditions. Among these behaviors are chaos, multistability, time series analysis periodicity and quasi periodicity which can manifest in the system in various situations. In Figs (10)–(12), we have explored the multistability and time series examination of the perturbed system (23) under various beginning constraints. System (23) appears to be especially vulnerable to chaotic initial conditions based on observations. Comprehending the characteristics of multistability and time series analysis, which is an essential constituent of intricate dynamic systems, helps facilitate the explanation and prediction of these system’s behaviours under diverse conditions.

**Fig 13:**
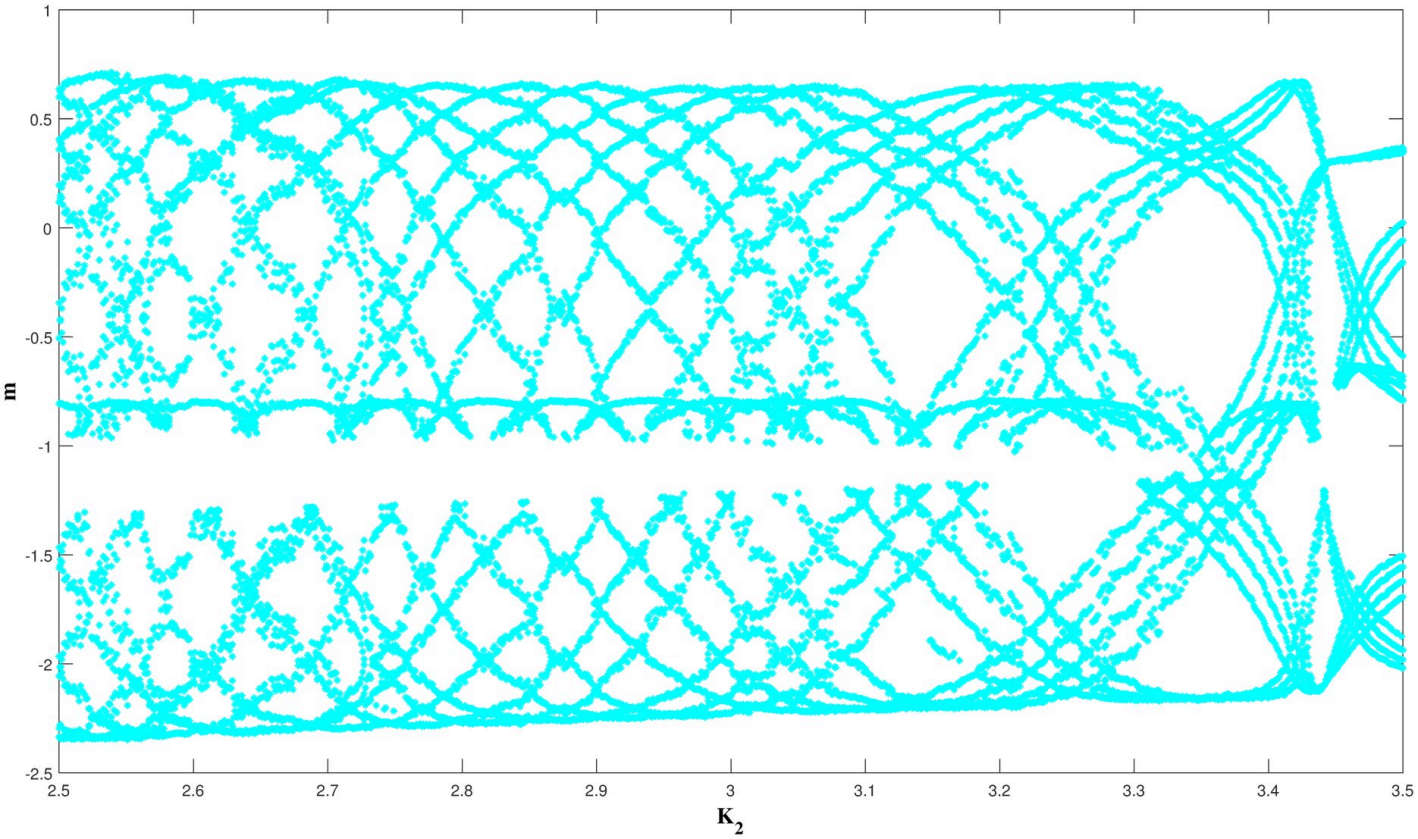
The study of chaotic behavior for system (23) through bifurcation diagram: Bifurcation *m* vs *K*_2_ at *K*_1_ = 3, *K*_3_ = 5.2, Ω = 3.7, *θ* = 1.8, and initial condition condition (0.4,0.4,0.4).

A visual representation that shows how a dynamical system behaves when a parameter is changed is known as bifurcation diagram. The magnitude or strength of the perturbation is the parameter of interest in the scenario of a dynamical system that is perturbed. One can learn more about the possible behaviours of the system, such as fixed points, chaos, or limit cycles, by looking at the bifurcation diagram. Bifurcation diagrams, especially, can be used to determine the key parameter values, such as the beginning of chaos or the shift from stable to unstable dynamics, at which the system experiences a qualitative change in behaviour. In (Fig 13) we explore the bifurcation diagram of the perturbed dynamical system (23) versus *m* vs *K*_2_ with beginning condition (0.4,0.4,0.4) and physical parameters such as *K*_1_ = 3, *K*_3_ = 5.2, Ω = 3.7, *θ* = 1.8.

## Conclusion

The *q* deformed Sinh-Gordon equation has been investigated by soliton structure and chaotic behaviour. The underlying equation is converted into an ordinary differential equation using the wave transformation technique. The new extended direct algebraic and modified auxiliary equation techniques have been effectively applied to obtain novel analytical traveling wave solutions. It is crucial to note that Sinh-Gordon equation solutions are achieved by utilizing hyperbolic, trigonometric, exponential, and rational functions. A variety of soliton structures have been produced for the resulting ordinary differential equation, taking into account different parameter values. Visual representations in both 2D and 3D forms are subsequently produced.

(Fig 1) depicts the kink-bright soliton structures for *U*_6_ by setting distinct temporal component values as *t* = 0, *t* = 1, and *t* = 2 and *α* = 0.2, *α* = 0.5, *α* = 0.8, respectively. (Fig 2) exemplifies the bright soliton structures for *U*_8_ using the same temporal parameter values. Dark soliton structures for *U*_16_ are portrayed in (Fig 3) for different temporal component values as *t* = 0, *t* = 1, and *t* = 2 and *α* = 0.3, *α* = 0.5, and *α* = 0.7, respectively. In (Fig 4), bright-dark soliton structures are displayed for *U*_19_ by choosing different temporal parameter values as *t* = 0, *t* = 1, and *t* = 2, *α* = 0.2, *α* = 0.4, and *α* = 0.6, respectively. (Fig 5) represents kink soliton structures for *m*_3_ by selecting different temporal parameter values as *t* = 0, *t* = 1, and *t* = 2, *α* = −0.2, *α* = −0.5, and *α* = −0.8, respectively. Kink-peakon soliton structures for *m*_4_ are shown in (Fig 6) by picking different temporal parameter values as *t* = 0, *t* = 1, and *t* = 2, *α* = 0.2, *α* = 0.5, and *α* = 0.8, respectively.

Numerous chaos detecting tools, such as Lyapunov exponents, multistability and time series analysis and bifurcation diagram have been implemented. Lyapunov exponents in Figs (7)–(9) portrays the chaotic behavior of the model at various initial conditions. Moreover multistabiltiy analysis depicts Figs (10)–(12) the chaotic behavior of the model under consideration at distinct beginning constraints. Furthermore bifurcation diagram (Fig 13) illustrates the chaotic behavior of the investigated model. The findings illustrate the effectiveness of the suggested methods in exploring novel solutions for numerous NLPDE found across a variety of disciplines of applied nonlinear sciences.
