# Correction: Computation of soliton structure and analysis of chaotic behaviour in quantum deformed Sinh-Gordon model

**DOI:** 10.1371/journal.pone.0308186

**Published:** 2024-07-26

**Authors:** Adil Jhangeer, Farheen Ibraheem, Tahira Jamal, Muhammad Bilal Riaz, Atef Abdel Kader

There are errors in the author affiliations. The correct affiliations are as follows:

Adil Jhangeer^1,2^, Farheen Ibraheem^3^, Tahira Jamal^4^, Muhammad Bilal Riaz^1,5^, Atef Abdel Kader^6^

**1** IT4Innovations, VSB-Technical University of Ostrava, Ostrava-Poruba, Czech Republic, **2** Department of Mathematics, Namal University, 30KM Talagang Road, Mianwali 42250, Pakistan, **3** Department of Mathematics, Forman Christian College-A Chartered University-FCCU, Lahore, Pakistan, **4** Department of Mathematics, University of the Punjab, Lahore, Pakistan, **5** Department of Computer Science and Mathematics, Lebanese American University, Byblos, Lebanon, **6** College of Humanities and Sciences, Ajman University, Ajman, UAE.

The fourth and fifth authors, Muhammad Bilal Riaz and Atef Abdel Kader, should not have been attributed equal contribution to this work.
